# Neutrophil-to-Lymphocyte Ratio as a Promising Non-Invasive Biomarker for Diagnosis of Feline Idiopathic Cystitis in Cats

**DOI:** 10.3390/ani15223307

**Published:** 2025-11-17

**Authors:** Jingyi Yang, Xu Zhang, Wenkai Zhang, Yiwen Zhang, Lei Shi, Liwei Zeng, Meilin Qiao, Hao Shi

**Affiliations:** 1 College of Veterinary Medicine, China Agricultural University, Beijing 100193, China; 2 Zhangxu Veterinary Hospital, Hangzhou 310051, China; 3 China Agricultural University Veterinary Teaching Hospital, Beijing 100193, China

**Keywords:** feline idiopathic cystitis (FIC), feline lower urinary tract disease (FLUTD), neutrophil-to-lymphocyte ratio (NLR), diagnosis

## Abstract

Feline idiopathic cystitis (FIC) is a common chronic cystitis disease in cats, accounting for 55–65% of feline lower urinary tract disease (FLUTD). The common clinical symptoms of FIC include pollakiuria, periuria, hematuria, and dysuria. At present, the disease is mainly excluded by evaluating the results of blood, urine, and imaging examinations of diseased animals. There is still a lack of clear and objective indicators for the diagnosis of FIC in clinical practice. Neutrophil-to-lymphocyte ratio (NLR) can be used as an indicator to measure immune inflammatory response and neuroendocrine pressure. It is an economical, efficient, and simple calculation method. In this study, the correlation between NLR and FIC was analyzed by collecting the complete blood count data of FIC cats and healthy cats. The results of inter-group difference comparison showed that the LYMPH levels in the normal group were significantly higher than that in the FIC group (*p* < 0.001), and the NEUT levels and NLR levels were distinctly lower than those in the FIC group (*p* < 0.001). Spearman correlation analysis showed that there was a significant correlation between NLR and FIC (r = −0.8439, *p* < 0.0001). ROC analysis showed that NLR had high diagnostic accuracy in distinguishing healthy cats from FIC cats (AUC = 0.9872). In general, this study preliminarily confirmed that there was a significant correlation between NLR elevation and FIC, emphasizing the prospective utility of NLR as a promising biomarker for diagnosis in FIC. Because of the lack of uniform diagnostic criteria for FIC, NLR may provide important help in the diagnosis process.

## Simple Summary

Feline idiopathic cystitis (FIC) is a chronic disorder of the lower urinary tract in cats. At present, the disease is mainly excluded by evaluating the results of blood, urine, and imaging examinations of diseased animals. There is still a lack of clear and objective indicators for the diagnosis of FIC in clinical practice. In this study, the correlation between NLR and FIC was analyzed by collecting the complete blood count data of FIC cats and healthy cats. The results of the inter-group difference comparison showed that the NLR levels in the normal group were distinctly lower than that in the FIC group (*p* < 0.001). Spearman correlation analysis showed that there was a significant correlation between NLR and FIC (r = −0.8439, *p* < 0.0001). ROC analysis showed that NLR had high diagnostic accuracy in distinguishing healthy cats from FIC cats (AUC = 0.9872). Therefore, the NLR parameter holds the potential to serve as a non-invasive biomarker of FIC.

## 1. Introduction

Feline idiopathic cystitis (FIC) is a chronic cystitis disease commonly seen in cats, accounting for approximately 55% to 65% of feline lower urinary tract disease (FLUTD) cases [1]. The exact cause of this condition is still unclear. Affected animals typically exhibit chronic irritative urinary symptoms, without evidence of bacteriuria and pyuria [2]. Research indicates FIC development is influenced by multiple factors, including genetics, obesity, diet, stress, and hormonal changes following neutering [3]. Additional research indicates that the primary pathophysiological manifestations of FIC result from central nervous system stimulation, chronic stress-induced adrenal insufficiency, or alterations in urinary bladder permeability [4,5]. Reports suggest that compared to other urinary tract diseases, FIC represents a more complex condition involving multiple organs and systems [6,7,8].

Common clinical symptoms of FIC include pollakiuria, periuria, hematuria and dysuria. Currently, diagnosis is primarily made through exclusionary methods: evaluating clinical signs and medical history, conducting a comprehensive physical examination, blood analysis, urinalysis, urine culture, and urinary tract imaging. FIC is typically confirmed after ruling out urinary tract infections, urolithiasis, anatomical abnormalities, behavioral issues, and neoplasms [7]. Unfortunately, there is still a lack of uniform diagnostic criteria for FIC.

Previous investigations indicate that urinary N-acetyl-β-D-glucosaminidase (NAG) may be a potential biomarker for FIC [4]. The urothelium, which is a part of the bladder wall contains three layers: the basal, intermediate, and superficial apical layer. Superficial urothelial cells play a pivotal role in maintaining the bladder’s protective barrier, which primarily relies on a glycosaminoglycan (GAG) layer covering the luminal surface of the urothelium [9]. Any factor that disrupts the structural or functional integrity of GAGs, or directly damages the urothelium, can compromise the barrier’s function [10]. Previous studies have shown that cats with feline idiopathic cystitis (FIC) exhibit reduced urinary concentrations of total GAGs [11] and elevated urinary protein-to-creatinine (UPC) ratios compared with healthy cats [4]. N-acetyl-β-D-glucosaminidase (NAG) has been implicated in the degradation of circulating GAGs, and proteinuria is recognized as one of the characteristic features of FIC [12,13]. Accordingly, alterations in urinary NAG concentration may influence the GAG layer lining the bladder and reflect underlying pathological changes in affected cats [4].

In 2001, Zahorec and colleagues proposed a novel indicator for assessing immune-inflammatory responses and neuroendocrine stress, later termed the neutrophil-to-lymphocyte ratio (NLR) [14]. This parameter is derived by dividing the neutrophil count (NEUT#) by the lymphocyte count (LYMPH#) and can be obtained through a simple and readily accessible complete blood count (CBC). Consequently, it represents an economical, efficient, and straightforward computational method. Studies have demonstrated that the NLR parameter serves as an inflammatory marker associated with chronic diseases [14], and it is considered a simple indicator for assessing an individual’s inflammatory status [15].

Neutrophilia and lymphopenia are recognized as fundamental indicators of innate immune activation in the context of systemic inflammation [16]. Retrospective findings have identified a close link between the neutrophil-to-lymphocyte ratio (NLR) and inflammatory pathophysiological processes in cats [17]. In felines affected by chronic kidney disease (CKD), a progressive condition marked by sustained inflammatory activity, NLR values tend to rise substantially in advanced stages when compared with both early CKD and healthy cohorts [18]. Investigations into systemic inflammatory disorders have demonstrated that NLR values are notably elevated in cats suffering from systemic inflammatory response syndrome (SIRS) or sepsis relative to clinically normal counterparts. Elevated NLR has also been independently associated with higher mortality rates in these populations [19]. Furthermore, retrospective evidence indicates that cats experiencing blunt trauma often exhibit increased NLR, suggesting its potential utility as a marker for assessing injury severity [20]. Within the scope of feline neoplastic conditions, markedly greater preoperative NLR values have been observed in individuals diagnosed with infiltrative injection-site sarcomas, fibrosarcomas, and those presenting with local tumor recurrence following surgical removal [21]. In addition, heightened NLR levels have been correlated with a significantly increased likelihood of tumor-associated death [22].

Previous studies indicate that blood cortisol levels and neutrophil counts increase under stress conditions [23]. Elevated cortisol may result from physical and mental stresses and sympathetic nervous system activity [24], while increased neutrophils may correlate with higher blood cortisol levels [25]. Cortisol influences multiple cellular and physiological functions to maintain organismal homeostasis. During physical load, cortisol primarily functions as an effector of the general stress response. Concurrently, it suppresses inflammation and certain immune reactions while mobilizing neutrophils [23]. Therefore, an elevated NLR may be stress related. Furthermore, human medical studies indicate that NLR accurately predicts conditions such as sepsis and infectious diseases [26], as well as cancer [27]. Simultaneously, this indicator holds potential as a non-invasive diagnostic marker and symptom indicator for human interstitial cystitis (IC) [28]. FIC is a chronic cystitis disease similar to IC [29]. We speculated that the NLR parameter may also hold potential in the early prediction of FIC. Therefore, this study collected complete blood count data from cats with FIC and healthy cats to statistically analyze NLR, investigate the correlation between NLR and FIC, and preliminarily explore the feasibility of the NLR parameter as a promising non-invasive biomarker of FIC. This aims to provide data support for the correlation between NLR and FIC in veterinary clinical practice and evaluate the potential of NLR as a non-invasive indicator of potential FIC.

## 2. Materials and Methods

### 2.1. Candidates

Data were collected from a randomly selected cohort of 100 cats treated at the China Agricultural University Veterinary Teaching Hospital and Zhe Jiang Zhangxu Veterinary Hospital from 2022 to 2025.

### 2.2. Methods

The basic information (including breed, age, gender, and body weight) and medical history of each cat were collected, and their physical examination, complete blood count, and blood biochemistry results were recorded. We also collected urinalysis, urine culture, X-ray examination, and ultrasound examination results from the cats with FIC. An outline of the case definitions and the criteria applied for their selection [2,4,29] is presented in Table 1 and Table 2.

In addition, all blood samples were collected in a quiet, cat-specific sampling room, equipped with pheromones to enhance emotional comfort. Blood was drawn using standardized techniques from either the cephalic or saphenous vein of each animal and promptly sent for analysis.

In all included cats, hematological analyses were performed with the ProCyte Dx Hematology Analyzer (IDEXX, Westbrook, ME, USA). Blood smear examinations were also performed to validate the results obtained from the instrument analysis. Moreover, urine samples were cultured on blood agar and MacConkey agar to assess bacterial growth. Routine urinalysis was conducted to measure key physicochemical and microscopic parameters, including color, specific gravity, occult blood, protein, potential of hydrogen (pH), epithelial cells, crystals, glucose, ketones, urobilinogen, and other parameters.

All samples were divided into FIC and normal groups, with 50 samples in each group. The age, body weight, LYMPH levels, and NEUT levels were counted, and NLR was calculated.

Continuous variables following a normal distribution were expressed as mean  ±  standard deviation (SD), whereas non-normally distributed variables were presented as median (IQR). The Shapiro–Wilk test (S-W test) was used to detect the normality of the data. The Mann–Whitney U test (U test) and Chi-square test were used to detect the differences between groups. Spearman correlation analysis was used to evaluate the relationship between NLR and FIC. The ROC analysis was used to evaluate the diagnostic efficacy of NLR in distinguishing FIC cats from healthy cats. SPSS27 software was used for analysis.

## 3. Results

Breeds of cats with FIC includes domestic shorthair or longhair (*n* = 14), British Shorthair (*n* = 12), American Shorthair (*n* = 10), Ragdoll (*n* = 7), Garfield (*n* = 2), Exotic Shorthair (*n* = 2), Scottish Fold (*n* = 2), and Bengal (*n* = 1). The breed of normal cats was shorthair or longhair (*n* = 20), British Shorthair (*n* = 10), American Shorthair (*n* = 5), Ragdoll (*n* = 7), Garfield (*n* = 2), Devon Rex (*n* = 3), Maine Coon (*n* = 2), and Siamese (*n* = 1). No significant breed predispositions were found (*p* = 0.127).

Of the cats with FIC, 49 (98%) were males and 1 (2%) was female. Corresponding values for the normal group were 18 (36%) and 32 (64%), demonstrating a significant sex-associated predisposition (*p* < 0.001).

The LYMPH levels were significantly higher in the normal group than the FIC group (*p* < 0.001). On the contrary, the NEUT levels and NLR levels were distinctly lower than those in the FIC group (*p* < 0.001). At the same time, the body weight of the FIC group [5.00 (1.30) vs. 3.40 (1.40)] was distinctly higher. No significant differences were found in age (*p* = 0.053) (Table 3).

**Figure 1:**
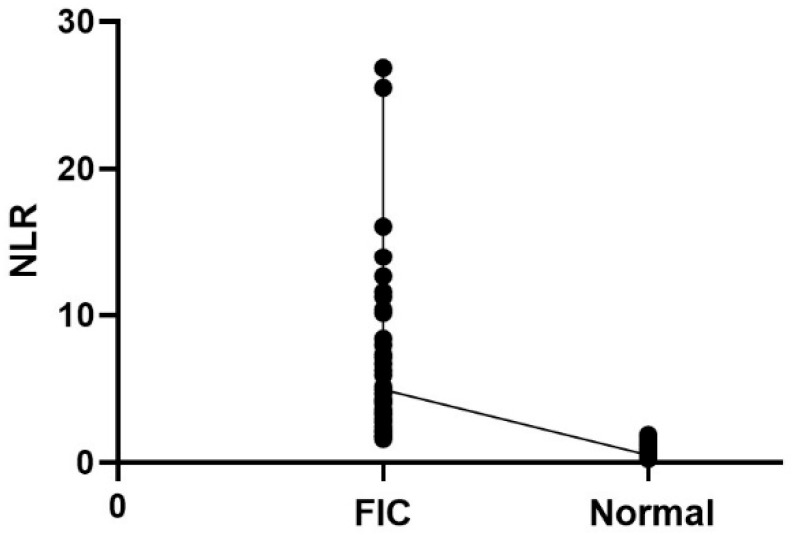
Correlation scatterplot of NLR with FIC.

We also performed Spearman correlation analysis to assess the correlation between NLR and FIC (Table 4). The results showed that there was a significant correlation between NLR and FIC (r = −0.8439, *p* < 0.0001) (Figure 1).

**Figure 2:**
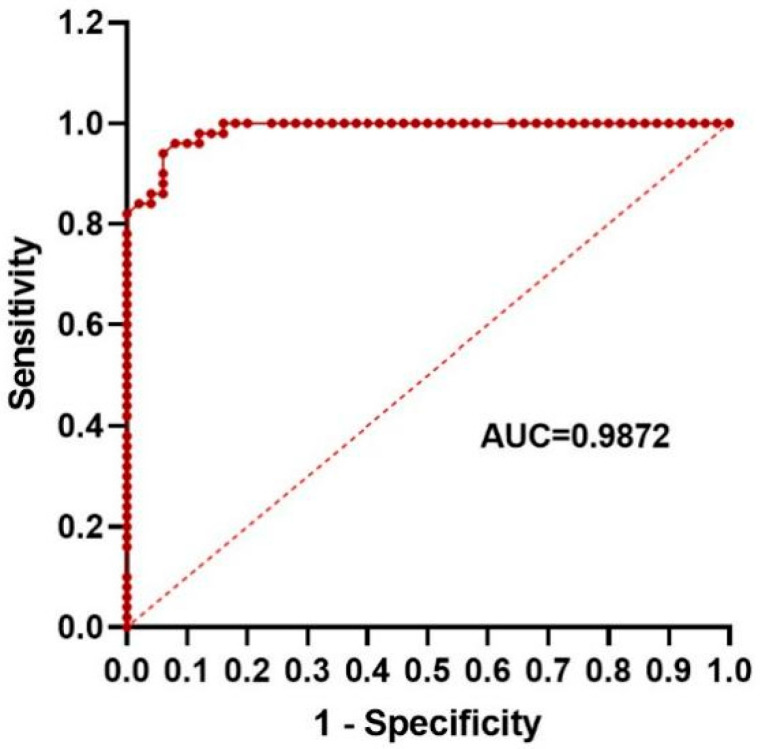
Receiver operating characteristic (ROC) curve of NLR.

We also used ROC analysis to evaluate the diagnostic efficacy of NLR in distinguishing FIC cats from healthy cats (Table S1). The results showed that when distinguishing FIC cats from healthy cats, the AUC value of NLR was 0.9872 (Figure 2), and the diagnostic accuracy was high. The maximum Youden index was 0.88, and the corresponding cutoff value was 1.785 and 1.795 (Table S1). As an early diagnostic indicator, 1.795 with higher sensitivity can be selected as the diagnostic cutoff value. This further suggests that the NLR parameter has a good potential to become a biomarker for diagnosis of FIC.

## 4. Discussion

This study showed that compared with healthy cats, FIC cats showed a significant increase in NLR and body weight. And there is a significant sex-associated predisposition in FIC. Through Spearman analysis, we determined that NLR was significantly correlated with FIC. Through ROC analysis, we determined that NLR has a good diagnostic utility in distinguishing FIC cats from healthy cats. These findings suggest that an elevated NLR holds a great potential to be a non-invasive biomarker of possible FIC.

Neutrophils play a crucial role in innate immune responses, performing fundamental functions such as phagocytosis and releasing various cytokines. They also release reactive oxygen species and a series of peptides that aid in forming extracellular traps, but these can adversely affect the bladder [30]. Inflammatory responses can cause neutrophilia, and tumor necrosis factor triggers lymphocyte apoptosis during the early stages of inflammation [31]. Numerous studies indicate that stress may contribute to FIC development. Short- or long-term exposure to abnormal external events and unpredictable stressors can induce tension and fear in cats, leading to FIC [8,32,33]. Concurrently, lymphopenia with neutrophilia is a symbol of stress [24]. Therefore, stressed animals exhibiting reduced neutrophil percentages and increased lymphocyte percentages [34]. Consequently, NLR serves as an inexpensive, simple, rapid-response, and readily accessible indicator of stress and inflammation. With high sensitivity and low specificity, it effectively captures the complex interplay between stress and inflammation, reflecting the equilibrium between innate and adaptive immune responses [35]. The increase in NLR values is directly associated with diseases characterized by severe inflammation, stress, injury, trauma, or cancer. Our findings followed the same trend as previous studies, which reported that there is a significant association between FIC incidence and overweight status [8,32,36]. This phenomenon may be linked to the reduced activity commonly observed in overweight cats. Similarly, cats with cystitis often exhibit decreased mobility [37]. Such inactivity may be indicative of stress, as cats subjected to prolonged stress frequently show a reduction in exploratory and playful behaviors, along with an increased tendency to hide [38].

Overall, compared with currently utilized biomarkers, the neutrophil-to-lymphocyte ratio (NLR) represents a cost-effective, easily accessible, and readily calculable parameter, making its incorporation into clinical practice both practical and promising. The findings of this study indicate that NLR has the potential to serve as a non-invasive biomarker for feline idiopathic cystitis (FIC).

Despite these encouraging results, several limitations should be acknowledged. Although an association between FIC and elevated NLR was observed, a causal relationship cannot be firmly established. Future investigations employing prospective designs and incorporating serial NLR measurements may provide more definitive evidence regarding the diagnostic utility of NLR in FIC. Additionally, this study did not include cats diagnosed with urinary tract infections (UTIs); therefore, the ability of NLR to differentiate between FIC and UTI remains undetermined.

This study offers several notable strengths. To our knowledge, it represents the first investigation exploring the relationship between NLR and FIC, and the use of validated and widely accepted analytical methods in veterinary research enhances the robustness and reliability of the results.

## 5. Conclusions

In summary, this study identified an association between NLR elevation and FIC, emphasizing the potential of NLR as a biomarker for FIC. Given the current lack of definitive objective indicators for diagnosing FIC in veterinary practice, NLR may offer significant assistance in the diagnostic process.

## Figures and Tables

**Table 1:** Case definitions.

Feline Idiopathic Cystitis (FIC)	Normal
Presented for one or more of lower urinary tract signs (LUTS), including stranguria, pollakiuria, periuria, hematuria, or dysuria. A complete diagnostic workup—consisting of physical examination, urinalysis, urine culture, and abdominal ultrasonography in every animal—failed to determine a specific etiology for the observed LUTS.	Presented for annual wellness exam and vaccination.

**Table 2:** Selection criteria.

Inclusion criteria The results of both urinalysis and urine culture are obtainable for a sample obtained through ultrasound-guided cystocentesis.
Exclusion criteria Patients with chronic or systemic diseases unrelated to the urinary system, including diabetes and cardiomyopathy; Patients with other urinary system diseases, such as urinary tract infection, urolithiasis, congenital anomalies of the urinary tract, or neoplastic disease; and, Patients with behavioral disorders.

**Table 3:** Baseline characteristics for each group.

	Normal	FIC	*p*-Value
*n*	50	50	
Age	2.50 (2.10)	3.50 (3.75)	0.053
Weight (kg)	3.40 (1.40)	5.00 (1.30)	<0.001
NEUT# (10^9^/L)	3.59 (2.24)	8.38 (8.94)	<0.001
LYMPH# (10^9^/L)	3.87 (2.67)	1.85 (1.49)	<0.001
NLR	0.98 (0.58)	4.09 (4.81)	<0.001

The age, body weight, LYMPH#, NEUT#, and NLR of the FIC group and the normal group did not conform to the normal distribution and were presented as median (IQR). Values were calculated using a u-test. NEUT#, neutrophil count; LYMPH#, lymphocyte count; NLR, neutrophil-to-lymphocyte ratio; FIC, feline idiopathic cystitis.

**Table 4:** Correlation coefficients between FIC with NLR.

	Normal	FIC
*n*	50	50
NLR	0.98 (0.58)	4.09 (4.81)
Spearman r	−0.8439	
95% confidence interval	−0.8936 to −0.7738	
*p*-value	<0.0001	

Data were presented as median (IQR). NLR, neutrophil-to-lymphocyte ratio; FIC, feline idiopathic cystitis.

## Data Availability

The original contributions presented in the study are included in the article/Supplementary Materials; further inquiries can be directed to the corresponding author.

## Supplementary Materials

The following supporting information can be downloaded at: https://www.mdpi.com/article/10.3390/ani15223307/s1, Table S1: The analysis of ROC.
