# Supplementary material for: Neutrophil-to-Lymphocyte Ratio as a Promising Non-Invasive Biomarker for Diagnosis of Feline Idiopathic Cystitis in Cats
Source: Animals (Basel). 2025 Nov 17;15(22):3307. doi: 10.3390/ani15223307 (PMC12649334; doi:10.3390/ani15223307)
Supplement: Supplementary file 1 [file animals-15-03307-s001.zip › animals-3958847-supplementary.pdf]

Table S1. The analysis of ROC

NLR	Sensitivity	Specificity	Youden index
< 0.2800	0.02	1	0.02
< 0.3650	0.04	1	0.04
< 0.4350	0.06	1	0.06
< 0.4650	0.08	1	0.08
< 0.4900	0.1	1	0.1
< 0.5050	0.16	1	0.16
< 0.5300	0.18	1	0.18
< 0.5600	0.2	1	0.2
< 0.5800	0.22	1	0.22
< 0.6200	0.24	1	0.24
< 0.6550	0.26	1	0.26
< 0.6650	0.28	1	0.28
< 0.6850	0.3	1	0.3
< 0.7200	0.32	1	0.32
< 0.7600	0.34	1	0.34
< 0.7850	0.36	1	0.36
< 0.8100	0.38	1	0.38
< 0.8350	0.42	1	0.42
< 0.8600	0.44	1	0.44
< 0.9000	0.46	1	0.46
< 0.9400	0.48	1	0.48
< 0.9750	0.5	1	0.5
< 0.9950	0.52	1	0.52
< 1.005	0.54	1	0.54
< 1.025	0.56	1	0.56
< 1.045	0.58	1	0.58
< 1.060	0.6	1	0.6
< 1.075	0.62	1	0.62
< 1.085	0.64	1	0.64
< 1.105	0.66	1	0.66
< 1.140	0.68	1	0.68
< 1.165	0.7	1	0.7
< 1.195	0.72	1	0.72
< 1.225	0.74	1	0.74
< 1.335	0.76	1	0.76
< 1.495	0.78	1	0.78
< 1.575	0.82	1	0.82
< 1.610	0.84	0.98	0.82
< 1.640	0.84	0.96	0.8
< 1.685	0.86	0.96	0.82
< 1.730	0.86	0.94	0.8

< 1.760	0.88	0.94	0.82
< 1.775	0.90	0.94	0.84
< 1.785	0.94	0.94	0.88
< 1.795	0.96	0.92	0.88
< 1.810	0.96	0.90	0.86
< 1.850	0.96	0.88	0.84
< 1.885	0.98	0.88	0.86
< 1.895	0.98	0.86	0.84
< 1.910	0.98	0.84	0.82
< 1.955	1	0.84	0.84
< 2.025	1	0.82	0.82
< 2.130	1	0.8	0.8
< 2.320	1	0.76	0.76
< 2.490	1	0.74	0.74
< 2.645	1	0.72	0.72
< 2.860	1	0.7	0.7
< 2.975	1	0.68	0.68
< 3.090	1	0.66	0.66
< 3.225	1	0.64	0.64
< 3.330	1	0.62	0.62
< 3.510	1	0.6	0.6
< 3.640	1	0.58	0.58
< 3.675	1	0.56	0.56
< 3.835	1	0.54	0.54
< 4.010	1	0.52	0.52
< 4.090	1	0.5	0.5
< 4.165	1	0.48	0.48
< 4.245	1	0.46	0.46
< 4.495	1	0.44	0.44
< 4.815	1	0.42	0.42
< 5.055	1	0.4	0.4
< 5.510	1	0.36	0.36
< 5.900	1	0.34	0.34
< 6.100	1	0.32	0.32
< 6.485	1	0.3	0.3
< 6.920	1	0.28	0.28
< 7.230	1	0.26	0.26
< 7.660	1	0.24	0.24
< 8.020	1	0.22	0.22
< 8.230	1	0.2	0.2
< 9.290	1	0.18	0.18
< 10.28	1	0.16	0.16
< 10.82	1	0.14	0.14
< 11.42	1	0.12	0.12

< 12.12	1	0.1	0.1
< 13.32	1	0.08	0.08
< 15.04	1	0.06	0.06
< 20.80	1	0.04	0.04
< 26.19	1	0.02	0.02
